# Dynamic characteristics and synergistic effects of ecosystem services under climate change scenarios on the Qinghai–Tibet Plateau

**DOI:** 10.1038/s41598-022-06350-0

**Published:** 2022-02-15

**Authors:** Yanyun Luo, Dewei Yang, Patrick O’Connor, Tonghua Wu, Weijing Ma, Lingxing Xu, Ruifang Guo, Jianyi Lin

**Affiliations:** 1 Key Lab of Urban Environment and Health, Institute of Urban Environment, Chinese Academy of Sciences, Xiamen, 361021 China; 2 University of Chinese Academy of Sciences, Beijing, 100049 China; 3 School of Geographical Sciences, Southwest University, Chongqing, 400715 China; 4 Centre for Global Food and Resources and School of Biological Sciences, University of Adelaide, Adelaide, 5005 SA Australia; 5 Cryosphere Research Station on the Qinghai-Tibet Plateau, State Key Laboratory of Cryospheric Science, Northwest Institute of Eco-Environment and Resources, Chinese Academy of Sciences, Lanzhou, 730000 China; 6 College of Earth and Environmental Sciences, Lanzhou University, Lanzhou, 730000 China; 7 Institute of Environmental Sciences (CML), Leiden University, PO Box 9518, 2300 RA Leiden, The Netherlands

**Keywords:** Ecosystem ecology, Climate-change ecology

## Abstract

The Qinghai-Tibet Plateau (QTP) supplies many ecosystem services (ESs) that maintain local and global pan-Asian populations and ecosystems. The effects of climate change on ES provision in the QTP will have far-reaching impacts on the region and the many downstream ecosystems and countries that depend on ESs from the "Third Pole". This study undertook a systematic assessment of ES provision, trade-offs and synergies between four ESs (raw material provision, water yield, soil retention, and carbon storage) under future climate scenarios (representative concentration pathway). The results show that: (1) the total amount of the four ESs on the QTP is predicted to increase from 1980 to 2100 for three climate change scenarios. (2) The spatial pattern of ESs on the QTP will not change significantly in the future, and the grassland and forest ESs in the central and southern regions are predicted to increase significantly. (3) The synergistic interactions among ESs were generally consistent at three spatial scales (10 km (pixel), county and watershed scales), but with more significant synergistic effects at the watershed scale. This demonstrates the necessity for the examination of scale-dependent ES dynamics and interactions. This study will supply a reference for further research on long-term ES assessments, especially the dynamic ES changes and the spatial scale dependency of the ES interactions, and provide evidence-based strategies for formulating ecosystem management on the QTP under climate change.

## Introduction

Ecosystem services (ESs) are a suite of benefits and welfare that human beings obtain from natural ecosystems and are essential to human well-being and the sustainability of human-natural systems^1^. Ecosystem services can be classified as supplying, supporting, regulating and cultural services^2^. Approximately 60% of global ESs are in decline due to climate warming and land cover change^3^. Changes in ESs are directly and indirectly affected by climate, soil, vegetation and land degradation^4^. Human-induced climate changes and land use change are reshaping ESs and ecological patterns and processes at an alarming rate^5^. Climate change affects ecological processes such as hydrological processes, material cycling and vegetation growth, which in turn affects ecosystem services, and makes it difficult to predict the trajectory of future ESs in climate sensitive geographies.

The effect of climate change on ecosystems has begun to be studied at the regional scale. Increased drought frequency is increasing the risk of grassland vulnerability in Mongolia^6^. Forest ESs in the Mediterranean region are predicted to suffer damage under a temperature increase of 2 °C^7^. Future climate change under specific representative concentration pathway (RCP) scenarios is predicted to increase flood risk in Western Africa^8^, change forest population structure in Great Britain^9^, and threaten the stability of Arctic wetlands^10^. Under specific RCPs, the Brazilian kelp bed ecosystem is at risk of being lost^11^, while the carbon storage capacity of forest ecosystems in South Asia is increased^12^. As seen from the above studies, the researchers predicted the impact of climate change on ES in each region based on climate change scenarios, and the impacts of climate change on ecosystems vary regionally. Nevertheless, most of their studies focused on specific ecosystems and ESs within the region, and the ES dynamics in the Qinghai-Tibet Plateau (QTP) simulated by the RCP scenario have not yet been sufficiently researched, making the potential impact of climate change on ESs still unclear.

The Qinghai-Tibet Plateau (QTP) is a priority region for ES trend analysis because it plays a vital role in maintaining regional and global ecological security, and is sensitive to global warming^13^. The rate of climate warming on the QTP has been approximately double the global average over recent decades resulting in unprecedented impacts on regional ecosystems^13,14^. There is a growing understanding of historical trends in ESs^15,16^, factors affecting ESs^17,18^, changing values of ESs^19^, and even regional ES development policies^20^. Analysis leading to predictions of future ESs in the QTP is needed to support policy and planning for a range of climate change scenarios where human well-being and ecosystem function are impacted.

With the deepening of research on ESs, rich quantitative tools, such as InVEST, ARIES, MIMES, EcoAIM, LUCI, EcoMetrix, ESR, Envision, EcoServ, and SAORES, have emerged^21^. The InVEST model developed by the Natural Capital Project has been widely used to evaluate ESs^22–25^. Compared with other models, the InVEST model is more suitable for the quantitative study of large-scale ecosystem services^22^. This integrated model can effectively evaluate multiple ecosystem services in space and time, and visualize the evaluation results spatially.

This is valuable because trade-offs and synergies between discrete ESs are influenced by the spatial heterogeneity of natural and social driving factors, and display complex and scale-dependent characteristics^2^. Most studies of the trade-offs and synergies between ESs are conducted at a single scale^26–28^, leaving the relationships of ESs at multiple scales uncertain or only partially considered. However, studies have shown that ESs and their functional relationships are affected by the spatial heterogeneity of natural and social driving factors, presenting complex characteristics of cross-scale dependence^2,29,30^. Conclusions at one scale may not be applicable to another^29^. Understanding trade-offs and synergies between ESs at multiple scales is essential for decision-making where scale is a critical social and political determinant of the decision-making process (e.g. national, regional, and local government decisions).

The objectives of this study are to predict the changing spatial and temporal trends in ESs in the QTP region under future climate scenarios and measure the trade-offs and synergies between ESs at different scales. This study (1) simulates the values of four ESs, i.e. Water yield(WY), soil retention(SR), carbon storage(CS), and raw material provision(RMP) on the QTP using the InVEST model under the RCP2.6, RCP4.5 and RCP8.5 scenarios; (2) analyses the temporal trends and spatial differences in the four ESs under the three RCP scenarios from 1980 to 2100; and (3) assesses the trade-offs and synergies between different ESs at the 10 km (pixel), county and watershed scales in the QTP region.

## Results

### Climate and land use changes under RCP scenarios

#### Climate change under RCP scenarios

**Figure 1:**
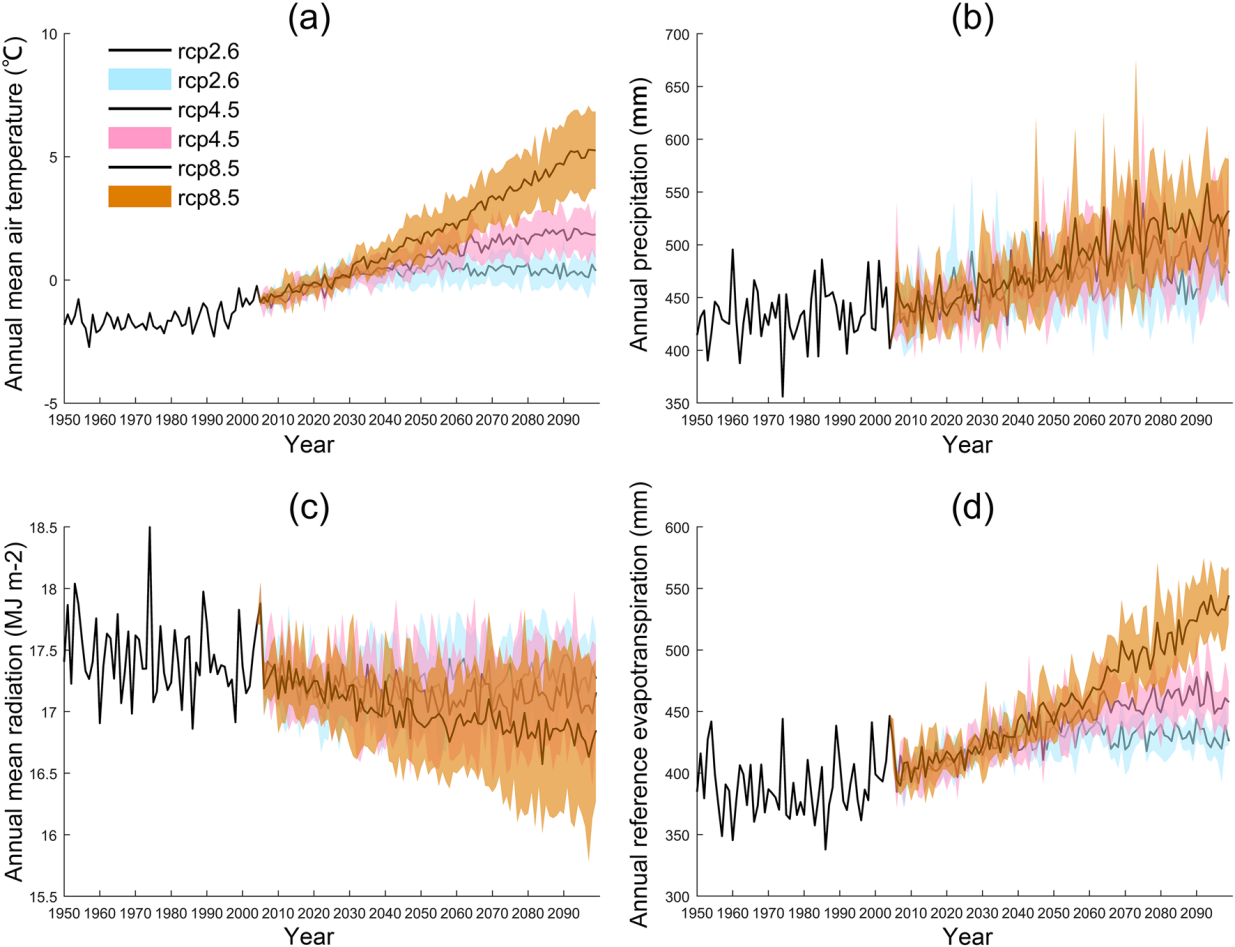
Climate change under different RCP scenarios (**a**). annual mean air temperature, (**b**). annual precipitation, (**c**). annual mean radiation, (**d**). annual reference evapotranspiration, the shaded area represents the standard deviation in five GCMs).

The climate change under the three RCP scenarios indicates that the QTP region will have a warming and humidification trend (Fig. 1). The annual mean temperature in the QTP region increased from − 1.56 °C in the baseline period(1950–2005) to 0.61(± 0.37), 1.84(± 1.01) and 5.26(± 1.58) °C in 2100 under the RCP2.6, RCP4.5 and RCP8.5 scenarios, respectively. The standard deviation of the annual mean temperature of the five GCMs under the RCP8.5 scenario is the largest. The annual precipitation increased from 433.13 mm in the baseline period to 473.15(± 31.11), 514.71(± 75.17) and 532.22(± 48.7)mm in 2100 under the RCP2.6, RCP4.5 and RCP8.5 scenarios, respectively. The standard deviation of the annual mean precipitation of the five GCMs under the RCP4.5 scenario is the largest. The annual mean radiation decreased from 17.44 MJ m^-2^ in the baseline period to 17.27(± 0.38), 17.16(± 0.38) and 16.85(± 0.58)MJ m^-2^ in 2100 under the RCP2.6, RCP4.5 and RCP8.5 scenarios, respectively. The annual reference evapotranspiration increased from 388.73 mm in the baseline period to 425.91(± 3.59), 457.62(± 16.32) and 544.34(± 22.94)mm in 2100 under the RCP2.6, RCP4.5 and RCP8.5 scenarios, respectively.

#### Land use change under the RCP scenarios

**Figure 2:**
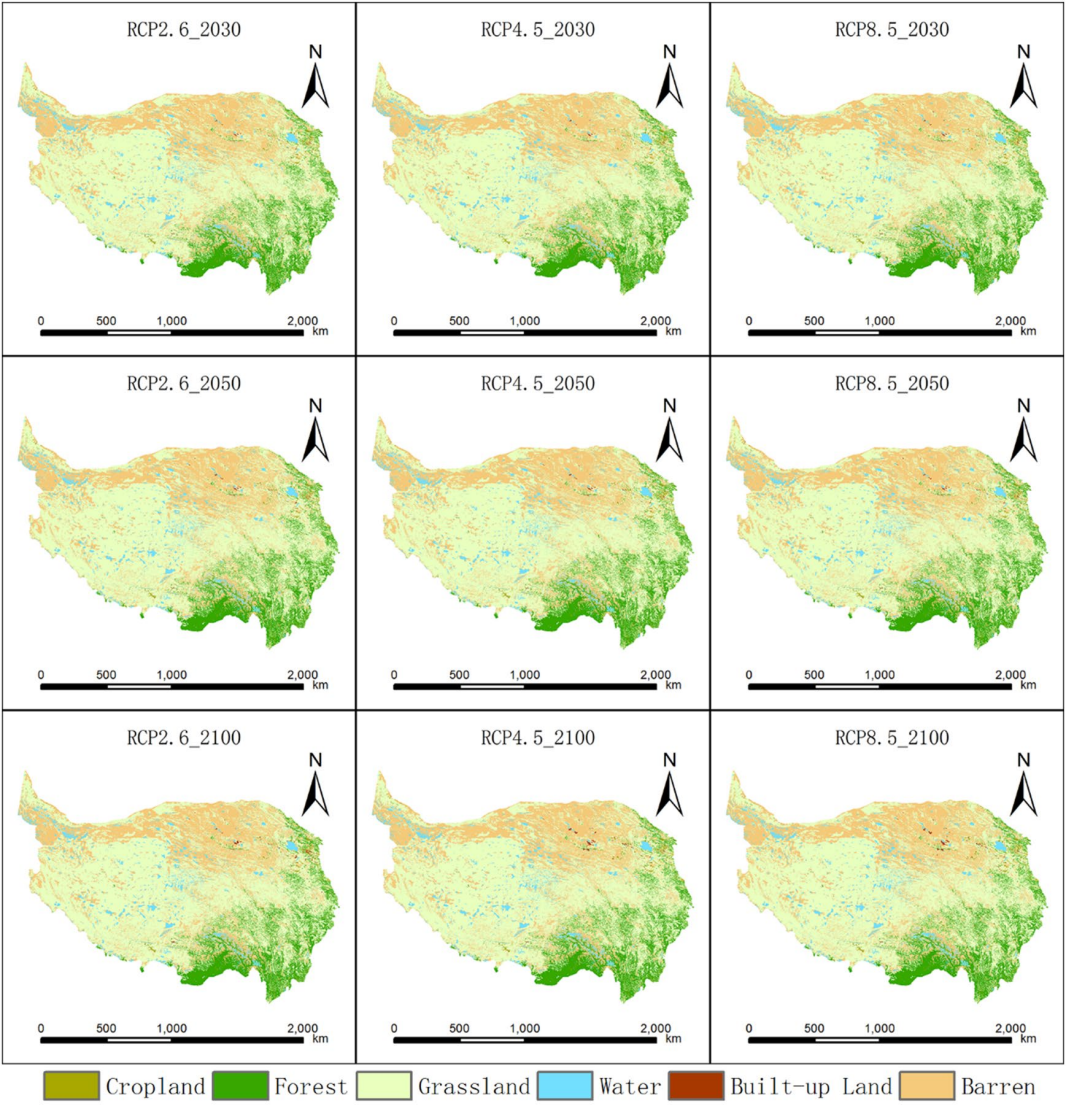
Land use map of the QTP region under the RCP2.6, RCP4.5 and RCP8.5 scenarios. The map was created using ArcMap 10.2, URL: http://www.esri.com.

The intensity of urbanization in the QTP region is low. The built-up area increased from only 1137 km^2^ in 1980 to 2302 km^2^ in 2015, and the area of cropland increased only from 18,604 km^2^ in 1980 to 18,945 km^2^ in 2015 (Table S3). Grassland is the dominant land use type in the QTP region and is mainly distributed in the central part of the plateau. There is a large area of barren land in the northern part of the plateau, and forest is mainly distributed in the eastern and southeastern parts of the plateau (Figure S1). The Future Land Use Simulation (FLUS) model validation shows that the overall accuracy and kappa coefficients are 95.09% and 94.11%, respectively. Under different RCP scenarios, the spatial pattern of land cover and the area of different land use types in the QTP have no significant differences. In the future, the area of forestland and water on the QTP will increase slightly due to regional climate warming and humidification. Moreover, the grassland area will decrease slightly due to the degradation of permafrost caused by climate warming (Fig. 2).

### Spatial distribution of ecosystem services

**Figure 3:**
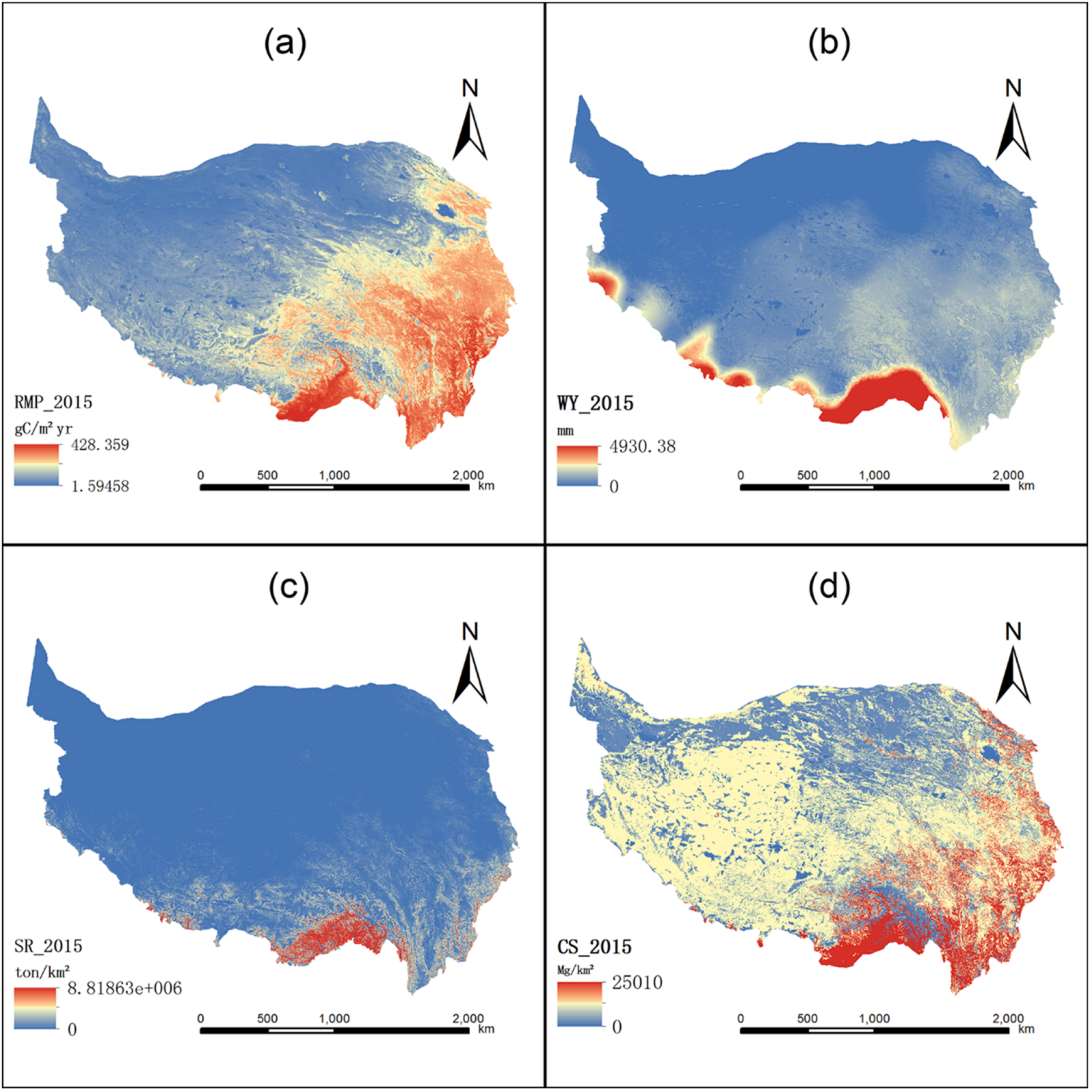
Spatial distribution of ESs in the QTP on 2015 ((**a**): raw material provision; (**b**): water yield; (**c**): soil retention; (**d**): carbon storage). The map was created using ArcMap 10.2, URL: http://www.esri.com.

The spatial pattern of ecosystem services on the QTP is high in the south and low in the north. The spatial distribution of ESs in the QTP region is highly correlated with natural characteristics (see Fig. 3). As the altitude increased, the four ESs on the QTP showed a decreasing spatial distribution from southeast to northwest in the observed period. This was caused by the gradient characteristics of elevation, precipitation and temperature. The four ESs were highest in the southeastern part of the plateau. This is due to the lowest elevation, milder climate conditions and abundant vegetation. The four ESs under the climate change scenarios of RCP2.6, RCP4.5 and RCP8.5 in 2030, 2050 and 2100 also show a similar spatial pattern of trending from high in the south to low in the north in 2015 (Figures S2–S5).

### Temporal changes in ecosystem services

#### Total amount of ecosystem services

**Figure 4:**
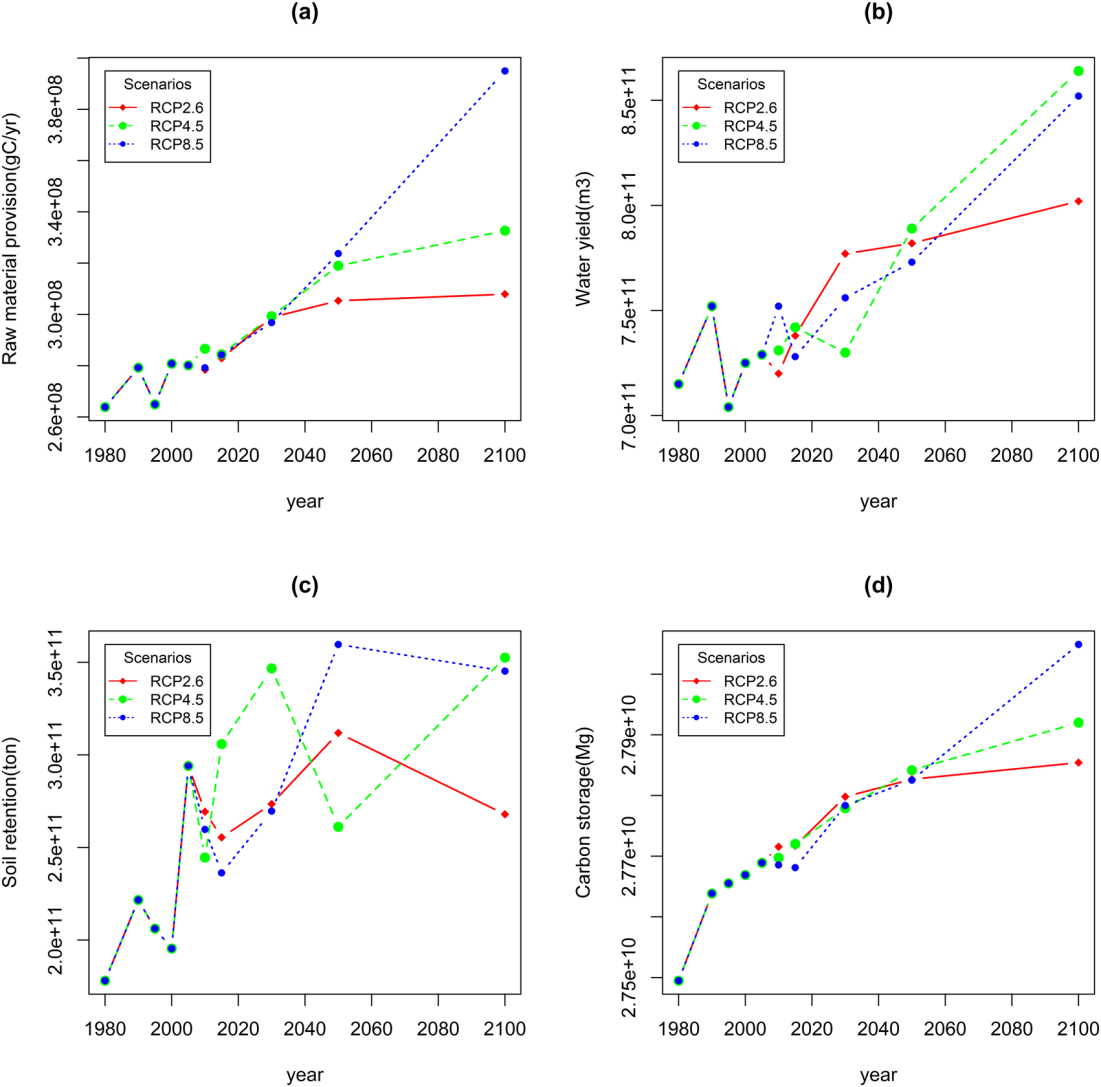
Changes in the total amount of ESs (**a**). raw material provision; (**b**): water yield; (**c**): soil retention; (**d**): carbon storage).

During 1980 ~ 2015, the changes in the overall magnitude of raw material provision and water yield were stable, while the soil retention and carbon storage increased slightly (Fig. 4). Compared with the baseline period, the four ESs increased significantly under the three RCP scenarios in 2030, 2050, and 2100 (Fig. 4). In the future, RMP services will increase the fastest under the RCP8.5 scenario, followed by RCP4.5, and the slowest under the RCP2.6 scenario due to improved hydrothermal conditions. Due to the increase in precipitation over the QTP caused by climate change, the WY service will increase in all three scenarios in the future, among which, it will increase significantly in the RCP8.5 and RCP4.5 scenarios. Changed climatic conditions will also lead to changes in land cover, leading to an increase in SR services. The increase in SR in RCP8.5 and RCP4.5 is higher than that in RCP2.6. Moreover, CS services will increase in the future under the three RCP scenarios.

#### Comparative trends in ecosystem service provisions

**Figure 5:**
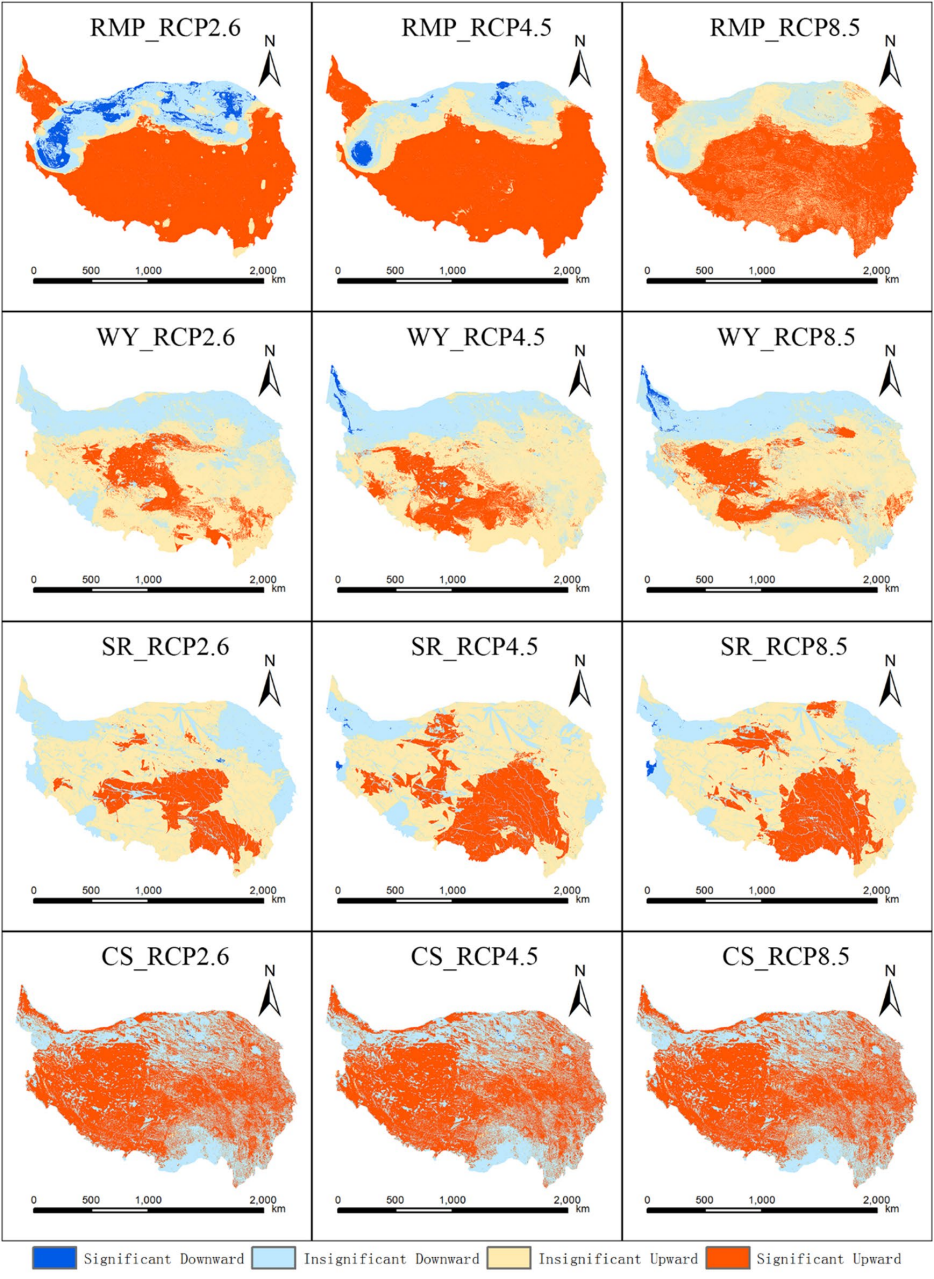
Spatial trend in ES provision under different RCP scenarios (*P* = 0.05). The map was created using ArcMap 10.2, URL: http://www.esri.com.

Under the three RCP scenarios, there were subregions with statistically significant changes in the four ESs (Fig. 5). The areas with a significant increase in water yield services were mainly concentrated in the central and southwestern parts of the QTP, and will gradually move westward in RCP2.6, RCP4.5 and RCP8.5. The area with a significant increase under the RCP4.5 scenario was the largest (Fig. 5). In addition, under the RCP4.5 and RCP8.5 scenarios, there were small areas in the northwestern QTP where water yield decreased significantly (Fig. 5). The areas where soil retention services increased significantly were mainly located in the central and southern parts of the QTP, and the areas where soil retention services increased significantly under the RCP2.6 scenario were the smallest (Fig. 5). On the QTP, the areas where raw material provision services increased significantly were distributed in the central, southern and northwestern parts of the QTP (Fig. 5). Under the RCP2.6 scenario, the area of raw material provision services that significantly decreased in the desert area of the northern QTP was the largest, followed by that under the RCP4.5 scenario, and the area with no significant decline under the RCP8.5 scenario (Fig. 5). The significant increase in CS services occurred mainly in the central QTP (Fig. 5). Under the RCP2.6, RCP4.5 and RCP8.5 scenarios, the slopes of the four ESs on the QTP were not significantly different. Water yield and soil retention services in the southern QTP increased significantly (Figure S6). The eastern and southeastern regions of the QTP experienced a significant increase in raw material provision services (Figure S6). The areas where carbon storage services increased significantly were located in the middle of the QTP (Figure S6).

### Ecosystem service interactions at different spatial scales

**Figure 6:**
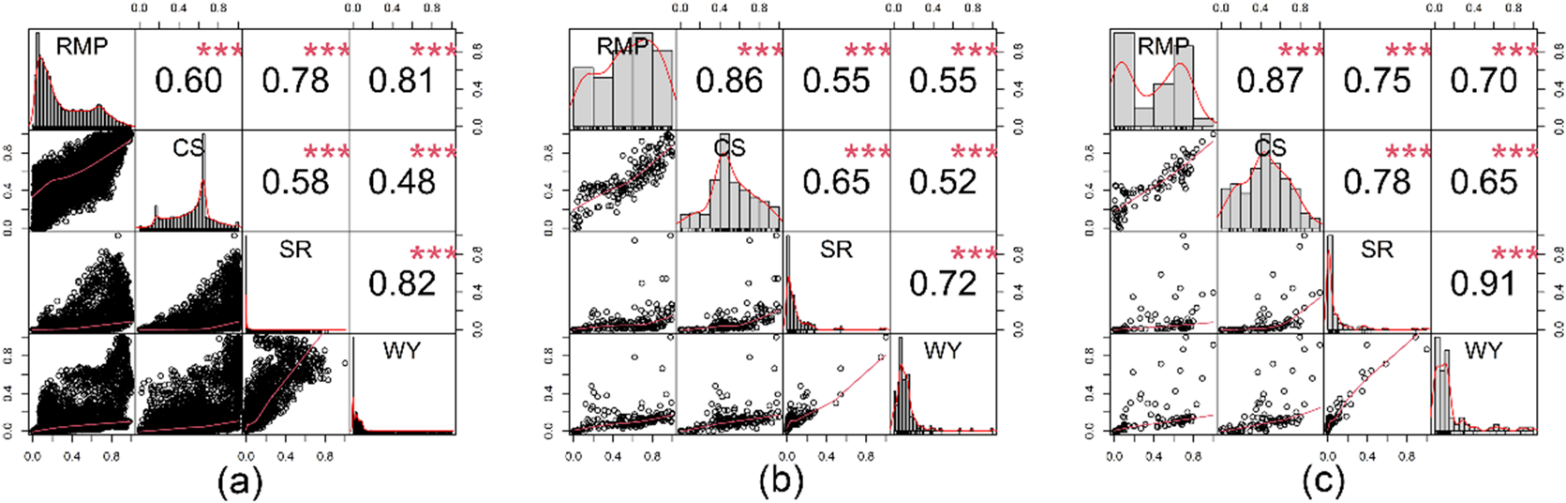
Trade-offs and synergies among ESs at the (**a**) 10 km, (**b**) county and (**c**) watershed scales in 2015.The number above the diagonal represents Spearman’s correlation coefficient. RMP: raw material provision; CS: carbon storage; SR: soil retention; WY: water yield. (*** for *p* < 0.001).

The Spearman correlation coefficient was calculated to evaluate the trade-offs and synergies of ESs on the QTP at the 10 km (pixel), county and watershed scales in 2015, 2030, 2050 and 2100. At the three scales, the four ESs showed statistically significant correlations (Fig. 6, S7–S9). Specifically, the synergistic effects of RMP-SR and RMP-WY were the strongest at the 10 km scale. At the watershed scale, RMP-CS, CS-SR, CS-WY, and SR-WY had the strongest synergistic effects. In addition, RMP-SR and RMP-WY had the weakest synergistic effects at the county administrative scale. In the three RCP scenarios in 2030, 2050 and 2100, the synergies between ESs did not change significantly after 2015(Figure S7–S9).

## Discussion

In this paper, we simulated the future trends of various ESs under three climate change scenarios on the QTP and analyzed their spatial patterns for extracting regulation strategies. First, the FLUS model is adopted to predict future land use. The results show that the area of forest and water will increase, while the area of grassland will decrease. The increase in forest area is mainly due to artificial afforestation and climate change^31^. The process of climate warming and humidification improves the hydrothermal conditions required for vegetation growth. The decrease in grassland area is mainly due to the transformation of part of grassland to forest and the degradation of permafrost, which results in the degradation of alpine meadows^31^. Moreover, the water area of the Qinghai-Tibet Plateau increases along with rising precipitation and glacial meltwater caused by climate change^31^. Second, the InVEST model and CASA model were combined with climate change scenarios to quantify trends in ESs in the QTP region. The spatial pattern of ESs does not change significantly under future climate change scenarios, and still shows a decreasing trend from southeast to northwest, which is consistent with the results of previous studies^32–34^. This is because the spatial distribution of land cover types and climate factors that affect the distribution pattern of ESs, such as temperature and precipitation, will not change significantly under future climate change scenarios. Under the three RCP scenarios, the ESs of the QTP showed an overall increasing trend from 1980 to 2100. In previous QTP studies, researchers found that the overall ESs have increased significantly in the past 25 years^15^, and the water yield services of the Shule River Basin in the northeastern QTP increased between 2001 and 2019^35^. In addition, studies have indicated that under future climate change scenarios, sandstorm prevention services are indirectly enhanced by climate change by altering the overall ecosystem pattern^36^. Our results confirm previous findings that the QTP has undergone a process of humidification and warming, and this process will continue^13,37,38^. The increase in ESs may be mainly due to warming and humidification in the QTP region. A predicted increase in temperature and precipitation will be conducive to the growth of vegetation, leading to a rise in the provision of the RMP service. However, in desert areas of the northern QTP, the RMP service shows a decreasing trend under the future three scenarios due to sparse vegetation. The increase in WY is mainly due to the rise in precipitation. Under RCP4.5 and RCP8.5, the arid climate and high evapotranspiration are predicted to result in a significant decrease in WY in the northwestern QTP. The increase in SR service is mainly due to changes in land cover and rainfall erosivity resulting from climate change. Under future climate change scenarios, climate warming and humidification will alleviate the energy constraint and prolong the growing season for alpine vegetation. In addition, the increase in CO2 concentration may produce a CO2 fertilization effect to promote the growth of vegetation, thereby increasing the CS service.

Our results indicate that future ESs will increase with the increase in radiative forcing in the RCP scenarios. Under future climate change scenarios, an increase in raw material provision services (such as forage and timber) may facilitate an improvement of residents’ livelihoods and regional sustainability. Additionally, the increase in water yield services has facilitated life and production in the region and provided water resources. However, it is worth noting that, as the source of many Asian rivers, the increase in water yield on the QTP may increase the risk of flooding in downstream areas, especially with extreme precipitation events. Therefore, changes in ESs under climate scenarios and their potential impacts on the QTP and its downstream areas should be considered in a spatially holistic climate change decision-making framework.

Studies have shown that ESs and their functional relationships are affected by the spatial heterogeneity of natural and social driving factors, presenting complex characteristics of cross-scale dependence^2,29,30^. This is because the biophysical connections behind ecosystem services are largely scale dependent^29^. However, findings from a single scale are not appropriate for extrapolation to multiple scales. This requires multiscale analysis to help us understand the trade-offs and synergies of ESs in detail, and to provide more comprehensive support for management decisions^39^. In this study, we analyzed the trade-offs and synergies among ESs at different administrative and natural scales, namely at 10 km (pixel), county, and watershed scales.

Our results show that at different scales and under three RCP scenarios, there are synergistic relationships among ESs in the QTP region (Fig. 6, Figure S6), indicating the stability of ES relationships in the region. This stability is consistent with the findings of other researchers in the Beijing-Tianjin-Hebei region^2^ and the Loess Plateau region^40^. Changes in the scale lead to changes in the strength of the observed synergies, which is consistent with the study of other researchers^41^, who suggest that potential trade-offs between ESs may be obscured by changes in spatial scales. The synergistic effect of RMP-SR and RMP-WY was the strongest at the 10 km pixel scale. The 10 km scale is more targeted to land cover types with high RMP service, such as forest and grassland, which also have high SR and WY services. At the watershed scale, RMP-CS, CS-SR, CS-WY, and SR-WY had the strongest synergistic effect. This may be related to the increased capability of characterizing hydrothermal conditions and material cycling ecological processes at the watershed scale than at the county scale and the 10 km pixel scale.

Synergies between ESs imply the possibility of the coincrease of multiple services, benefiting human well-being. Therefore, the goal of sustainable development can be achieved through a variety of ES portfolio management strategies. However, one certain ES policy on one spatial scale may not have the same effect at other scales^42^. Hence, it is essential to explore the trade-offs and synergies of a diversity of ES trends on multiple scales to balance and optimize ES provision^43^. Understanding the trade-offs and synergies between ESs at different scales, can be used to formulate appropriate ecological management policies.

Our study reveals the ESs of the QTP region under different climate change scenarios and provides a basis for climate change adaptation planning in the region. However, our analysis has some limitations. First, the objectivity and details of the results may be affected by the data sources used. The climate change dataset used in this study was the output of five CMIP5 GCMs, but different GCMs have different climate results for different regions, which may impact the results, and the 0.5° resolution of the climate change dataset may obscure details of the climate on the QTP. Second, studies have shown that changes in glaciers and permafrost caused by climate change may result in changes in the ecosystems in this area^44–46^. Unfortunately, due to a lack of comprehensive first-hand data, this study did not fully consider the impact of glacier and permafrost changes on the ESs of the QTP. Therefore, our results should be viewed in light of the need for additional research on direct ecosystem changes in QTP ESs. Third, we quantified only the four types of ESs under climate change scenarios and weighed the collaborative relationship between them due to data limitations for additional ESs. There is no comprehensive evaluation of ESs on the QTP. Finally, it is worth emphasizing that the changes in ESs on the QTP are closely related to the human well-being and sustainable development of downstream regions. Therefore, future research needs to comprehensively assess the impact of climate change on ESs from a cross-regional and multiscale perspective, and analyze the telecoupling^47^ of sustainability between the QTP and its downstream regions. This could advance the achievement of common sustainable development goals in multiple regions.

## Conclusions

As the “third pole in the world”, the QTP is highly sensitive to global climate change, particularly in its alpine ecosystem. However, comprehensive simulations of the trends in the supply of multiple ESs have rarely been conducted for multiple climate change scenarios. In this study, we combined biophysical models and spatially explicit models with RCP scenarios to simulate four ESs in the QTP during the historical period (1980–2015) and a future period (2030–2100), i.e., water yield, soil retention, carbon storage and raw material provision. During the study period, the QTP showed trends of increasing temperature and humidity under different climate scenarios, leading to an increased overall ES supply, and the increase in ES was significant under the RCP8.5 scenario. The forest and grassland areas in the southeastern QTP are the regions with high ES values. The significant increase in ES is mainly in the central part of the QTP, while the desert in the northern part of the QTP shows a significant decreasing trend in ES. In addition, the four types of ESs are bound in a synergistic relationship, with varying strength at different scales. Physically, the synergistic effect is strongest at the watershed scale.

The protection of forest and grassland ecosystems, and the impact of differences in scale on the synergistic effects of ESs should be emphasized in future management planning. This study is important for understanding the optimization of the pattern of ES at different scales, and formulating localized strategies to respond to climate change.

## Materials and methods

### Study area

**Figure 7:**
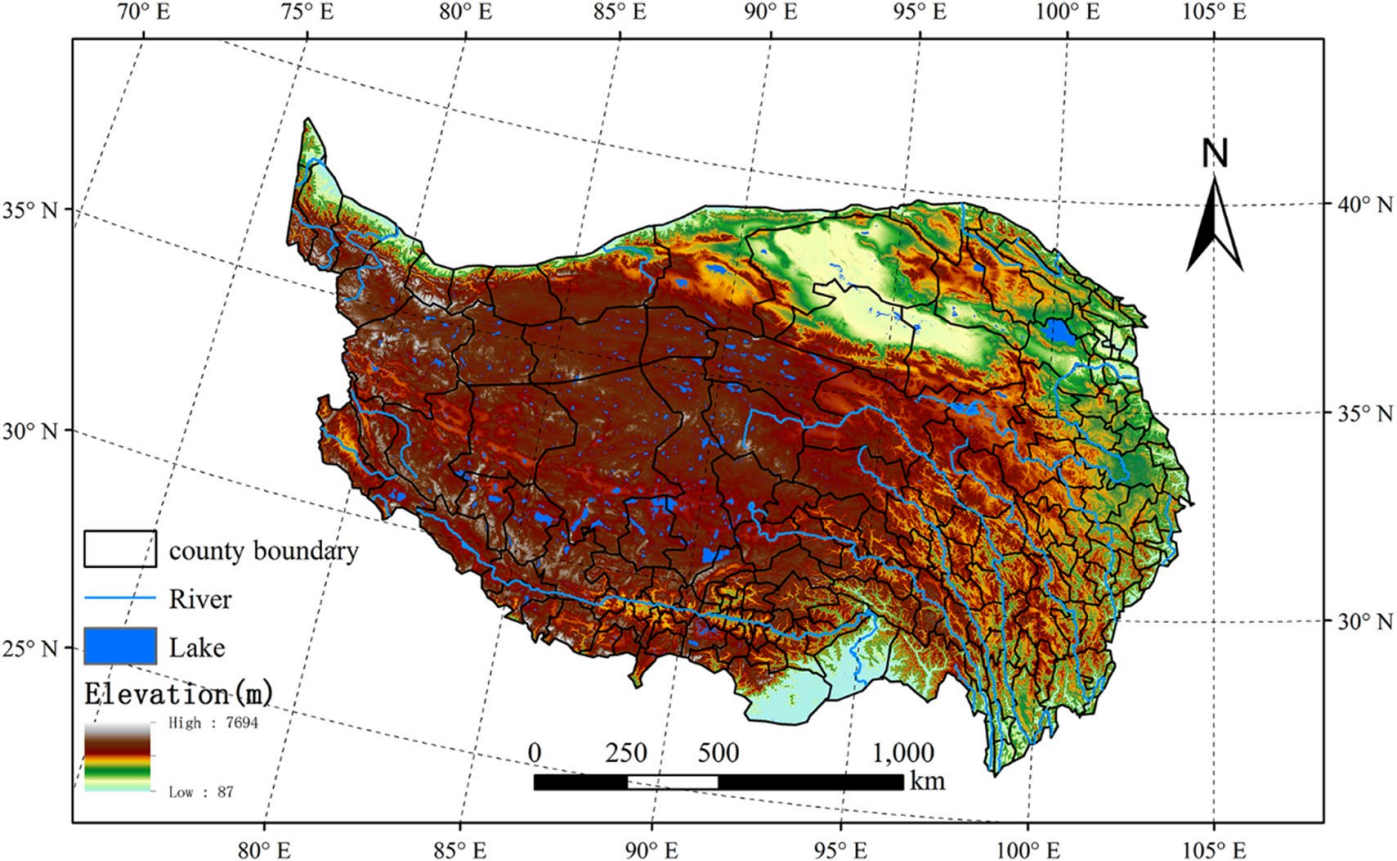
Geographical location of the QTP. The map was created using ArcMap 10.2, URL: http://www.esri.com.

The QTP is located in southwestern China (25° ~ 40°N, 75° ~ 103°E), with a total area of 2.5 million km^2^ and an average elevation above 4000 m (Fig. 7). The QTP is mainly covered with permafrost and grassland, with areas of glacier and desert^48^. The QTP, also known as the “Asian Water Tower”^49^, is the source of 13 major Asian rivers (e.g., the Indus, Ganges, Brahmaputra, Yangtze, and Yellow Rivers). The QTP has a clod, arid climate, with an annual average temperature below 0 °C and an annual mean precipitation of 400 mm. The seasonal distribution of precipitation is uneven, with most precipitation concentrated in the period June to September. There is a decreasing trend in precipitation from the southeast to the northwest of the plateau^50^. Known as the "Roof of the World" and "Third Pole", the QTP is also an area that is sensitive to global climate change, showing increasing warming and humidification in recent decades^51^. In addition, the QTP contains a diversity of ecosystems and fosters a historic ecological security barrier, which nurtures the development of animal husbandry and diverse cultures.

### Data sources

#### RCP scenarios and climate change dataset

The RCP scenarios released by the IPCC 5th Assessment Report^52^ supply a forecasting standard for climate change research. RCP values ranging from 2.6 to 8.5 reflect radiation forcing values in 2100 relative to the beginning of the Industrial Revolution in 1750^53^. Different radiative forcing scenarios represent different future climate scenarios. RCPs consist of one high-emission scenario (8.5 ${\text{W}} \cdot {\text{m}}^{ - 2}$, RCP8.5), two medium-emission scenarios (6.0 ${\text{W}} \cdot {\text{m}}^{ - 2}$, RCP6.0; 4.5 ${\text{W}} \cdot {\text{m}}^{ - 2}$, RCP4.5), and one low-emission scenario (2.6 ${\text{W}} \cdot {\text{m}}^{ - 2}$, RCP2.6)^54^. In this study, we adopted the RCP2.6, RCP4.5 and RCP8.5 climate change scenarios choosing RCP4.5 to represent the medium emission scenario in consideration of increasing activity through global initiatives in response to climate change. Specific descriptions of each scenario are shown in Table 1.

**Table 1:** The characteristics of each RCP scenario.

Scenarios component	Radiative forcing	CO_2_ concentration	Description
RCP2.6	2.6 ${\text{W}} \cdot {\text{m}}^{ - 2}$	420 ppm	Lowest level of economic and population growth and technological innovation; Promotes biomass energy utilization; Advocates forest restoration
RCP4.5	4.5 ${\text{W}} \cdot {\text{m}}^{ - 2}$	540 ppm	Sustainable economic, social and environmental development; Rapid development of low emission energy technology;
RCP8.5	8.5 ${\text{W}} \cdot {\text{m}}^{ - 2}$	940 ppm	Largest population; Low technological innovation; Slow energy improvement; Lack of policies to deal with climate change;

We adopted the climate change dataset outputs from five global circulation models(GCMs) (namely GFDL-ESM2M, HadGEM2-ES, IPSLCM5ALR, MIROC-ESM-CHEM, and NorESM1-M) within the fifth phase of the Coupled Model Intercomparison Project (CMIP5)^55^. The dataset outputs from GCMs were downscaled to a resolution of 0.5° and bias-corrected with Water and Global Change (WATCH) data (Integrated Project Water and Global Change, http:/www.eu-watch.org/data_availability)56. The baseline period of the dataset is 1950–2005 and the forecast period is 2006–2099.The climate change dataset included daily precipitation, air pressure, solar radiation, air temperature, maximum air temperature, minimum air temperature, wind speed, and relative humidity.

#### Auxiliary data

The auxiliary data for our research include the following. (1) The land use and land cover (LULC) map was obtained from the Resource and Environment Science and Data Center (RESDC), Chinese Academy of Sciences (https://www.resdc.cn) for 1980, 1990, 1995, 2000, 2005, 2010, 2015 and 2020 at a 1 km resolution. The LULC data have six major classes: cropland, grassland, forestland, water, built-up land and barren land. (2) The spatial distribution of soil type data, digital elevation model (DEM), watershed boundaries and normalized difference vegetation index (NDVI) data with a resolution of 1 km were obtained from the RESDC. (3) Soil physical and chemical property data (available soil water capacity, absolute depth to bedrock, silt content, clay content, sand content and soil organic carbon content) were obtained from the International Soil Reference and Information Centre (ISRIC Data Hub) (https://data.isric.org) with a 1 km spatial resolution. (4) During 1986–2005 and 1986–2098 (RCP2.6; RCP4.5; RCP8.5), the permafrost datasets in the Northern Hemisphere (10.12072/ncdc.CCI.db0032.2020) and the response of the alpine grassland ecosystem to climate change (RCP2.6, RCP4.5, and RCP8.5) in the permafrost region of the Qinghai-Tibet Plateau from 1981 to 2099 (10.12072/ncdc.CCI.db0006.2020) were provided by the National Cryosphere Desert Data Center (https://www.ncdc.ac.cn).

### Future land use simulation and validation

In this study, we used the Future Land Use Simulation model (FLUS) to simulate the LULC in 2030, 2050 and 2100 under the three RCP scenarios. This model was developed by^57^ and is available for download at (www.geosimulation.cn/flus.html). The FLUS model is an efficient land use simulation tool and has been widely used^58,59^. We selected the DEM, slope, precipitation, temperature, soil type, and permafrost distribution to calculate the suitability probability. Based on the land use transfer from 2010 to 2015, we calculated the total land use in 2030, 2050 and 2100 under three RCP scenarios by the Markov model. To validate the FLUS model, we set 2010 as the starting year and simulated the land use in 2015. The output results were compared with the real 2015 land use data, and we calculated the Kappa coefficient as follows:

1 $$\begin{array}{*{20}c} {Kappa = \frac{{P_{0} - P_{C} }}{{P_{P} - P_{C} }}} \\ \end{array}$$

where $P_{0}$ is the number of pixels converted correctly,$P_{C}$ is the correct number of pixels to be converted in the random case, and $P_{P}$ is the correct number of pixels to convert under ideal conditions.

### Assessment of ecosystem services under different RCP scenarios

This study assessed four ESs namely WY, SR, CS, and RMP, under climate change scenarios in 1980, 1990, 1995, 2000, 2005, 2010, 2015, and 2030 (short-term); 2050 (medium-term); and 2100 (long-term). We adopted the Integrated Valuation of Environmental Service and Tradeoffs (InVEST)^60^ model to assess the WY, SR, and CS ecosystem services. The InVEST model developed by the Natural Capital Project(www.naturalcapitalproject.org) is an effective model to evaluate ESs^61^ and is widely used in ES research on the QTP^22–25^. All spatial data were processed into a 1 km resolution and Albers projection by ArcGIS 10.2 before input into the InVEST model. The data requirements of the InVEST model and its processing are shown in Table S1. We use net primary productivity (NPP) to evaluate the RMP, and NPP can be used to represent the richness of biomass and the supply of organic materials. We adopted the Carnegie-Ames-Stanford Approach (CASA)^62^ model to estimate NPP.

#### Water yield

Water yield is a key ecosystem service. It refers to the annual quantity of water available for human use, as measured by the supply of surface water per unit area^63^. We adopted the InVEST 3.9.0 water yield model to estimate WY services in the QTP region. The water yield model is based on the water balance principle^64^. The biophysical parameter table required by the model is shown in Table S2. The parameters in the biophysical table come from the published literature^26,63,65^. The annual WY is calculated as follows:

2 $$\begin{array}{*{20}{c}} {{Y_{xj}} = \left( {1 - \frac{{AE{T_{xj}}}}{{{P_x}}}} \right){P_x}} \end{array}$$

where $Y_{xj}$ is the annual WY of land cover type j in pixel x; $P_{x}$ is the annual average precipitation of pixel x; and $AET_{xj}$ is the actual evapotranspiration of land cover type j in pixel x.

3 $$\begin{array}{*{20}c} {\frac{{AET_{xj} }}{{P_{x} }} = \frac{{1 + \omega_{x} R_{xj} }}{{1 + \omega_{x} R_{xj} + \frac{1}{{R_{xj} }}}}} \\ \end{array}$$

where $\omega_{x}$ is a dimensionless nonphysical parameter representing soil properties under natural climate conditions. The calculation method is as follows:

4 $$\begin{array}{*{20}c} {\omega_{x} = Z\frac{{AWC_{x} }}{{P_{x} }}} \\ \end{array}$$

where *Z* is a seasonal rainfall factor representing the regional precipitation distribution and other hydrogeological characteristics. The higher the *Z* value is, the less the seasonal constant *Z* affects the model results^66^. Since the QTP region belongs to the arid and cold climate zone in China, the *Z* value is set as 9. $AWC_{x}$ is the soil effective water content of pixel X, which is determined by the soil depth and physical and chemical properties. $R_{xj}$ is the Budyko dryness index, which is calculated as follows:

5 $$\begin{array}{*{20}c} {R_{xj} = \frac{{K_{xj} \cdot ET_{0} }}{{P_{x} }}} \\ \end{array}$$

where, $K_{xj}$ is the reference crop evapotranspiration and $ET_{0}$ is the reference evapotranspiration in pixel x. We adopted the modified Hargreaves method to calculate $ET_{0}$.

6 $$ET_{0} = 0.0013 \times 0.408 \times RA \times (T_{av} + 17) \times (TD - 0.0123P)^{0.76}$$

In the above formula, $T_{av}$ represents the average daily maximum temperature and minimum temperature, $TD$ represents the difference between the daily maximum temperature and minimum temperature, $RA$ represents astronomical radiation (MJm^-^^2^d^-1^) and *P* represents precipitation (mm/month).

#### Soil retention

Soil retention refers to the ability of various land cover types to prevent soil erosion. The InVEST 3.9.0 sediment delivery ratio (SDR) was employed to estimate SR services in the QTP region. The SDR model is based on the Revised Universal Soil Loss Equation (RUSLE)^67^, and the model is calculated as follows:

7 $$\begin{array}{*{20}c} {SR = R*K*LS - R*K*LS*C*P} \\ \end{array}$$

8 $$\begin{array}{*{20}c} {L = \left( {\frac{\gamma }{22.3}} \right)^{{\frac{\beta }{1 + \beta }}} } \\ \end{array}$$

9 $$\begin{array}{*{20}c} {\beta = \frac{{\sin \frac{\theta }{0.0896}}}{{\left[ {3.0\, *\,\left( {\sin \theta } \right)^{0.8} +\, 0.56} \right]}}} \\ \end{array}$$

10 $$\begin{array}{*{20}c} {S = 65.41*\sin^{2} \theta + 4.56*\sin \theta + 0.065} \\ \end{array}$$

where *SR* is the total amount of soil retention (tons ha^-1^ a^-1^), *LS* is the topographic factor, and *LS* is calculated from the slope length factor (*L*) and slope steepness factor (*S*). *C* is the vegetation and management factor. *P* is the support practice factor. *C* and *P* are shown in Table S2. *R* is the rainfall erosivity index(MJ mm ha^-1^ h^-1^ a^-1^), which was calculated via monthly precipitation^28^. *K* is the soil erodibility, which was calculated from the sand, silt, clay and organic soil moisture contents^68^. *R* and *K* are calculated as follows:

11 $$\begin{array}{*{20}c} {R = \mathop \sum \limits_{i = 1}^{12} \left( { - 1.5527 + 0.179P_{i} } \right)} \\ \end{array}$$

12 $$\begin{array}{*{20}c} {K = 0.1317*\left\{ {0.2 + 0.3*\exp \left[ { - 0.0256*SAN\left( {1 - \frac{SIL}{{100}}} \right)} \right]} \right\}} \\ {*\left( {\frac{SIL}{{CLA - SIL}}} \right)^{0.3} *\left( {1 - \frac{0.25*SOM}{{SOM + \exp 3.72 - 2.95*SOM}}} \right)} \\ {\quad \quad*\left( {1 - \frac{{0.7*1 - \frac{SAN}{{100}}}}{{\begin{array}{*{20}c} {1 - \frac{SAN}{{100}} + \exp \left( { - 5.51 + 22.9*\left( {1 - \frac{SAN}{{100}}} \right)} \right)} \\ \end{array} }}} \right)} \\ \end{array}$$

where *P*_*i*_ is the precipitation in month i. *SAN*, *SIL*, *CLA*, and *SOM* are the contents of sand, silt, clay and organic moisture, respectively. Other parameters are shown in Table S1.

#### Carbon storage

Carbon storage services refer to the carbon that ecosystems store in vegetation, soil and debris. The InVEST 3.9.0 carbon model uses a simple method to estimate CS based on land use data. The carbon pools in this model include four categories: aboveground carbon, belowground carbon, soil organic carbon and dead organic matter. This model simplifies the carbon cycle, and the change in carbon storage is mainly caused by change in land use69. The carbon pools for land use types were set according to published literature^70–72^. The carbon storage is calculated as follows:

13 $$\begin{array}{*{20}c} {{\text{C}}_{{{\text{total}}}} = C_{above} + C_{below} + C_{soil} + C_{dead} } \\ \end{array}$$

where ${\text{C}}_{{{\text{total}}}}$, $C_{above}$, $C_{below}$, $C_{soil}$ and $C_{dead}$ are the total carbon storage, aboveground carbon, belowground carbon, soil organic carbon and dead organic matter, respectively.

#### Raw material provision

Raw material supply refers to the organic matter provided by the ecosystem for human production and life, such as pasture and wood. In this study, RMP was quantified by the annual NPP. The NPP in the QTP region is calculated by the CASA model, which is a light use efficiency model driven by climate and remote sensing data^73,74^. The CASA model has been widely used to estimate NPP in terrestrial ecosystems^75,76^. In the CASA model, NPP is calculated as follows:

14 $$\begin{array}{*{20}c} {NPP\left( {x,t} \right) = APAR\left( {x,t} \right) \times \varepsilon \left( {x,t} \right)} \\ \end{array}$$

where, $APAR\left( {x,t} \right)$ is the photosynthetically active radiation(MJ m^-2^) absorbed by pixel x in month t, $\varepsilon \left( {x,t} \right)$ is the actual light energy utilization rate(gC MJ^-1^), and the $APAR\left( {x,t} \right)$ calculation method is as follows:

15 $$\begin{array}{*{20}c} {APAR\left( {x,t} \right) = SOL\left( {x,t} \right) \times FPAR\left( {x,t} \right) \times 0.5} \\ \end{array}$$

In the formula, $SOL\left( {x,t} \right)$ is the total solar radiation in pixel x in month t(MJ M^-2^); $FPAR\left( {x,t} \right)$ is the absorption ratio of photosynthetically active radiation by vegetation, which is determined by the normalized difference vegetation index (NDVI); and the constant 0.5 is the proportion of photosynthetically active radiation to the total radiation. $SOL\left( {x,t} \right)$ can be calculated by the solar shortwave radiation as follows:

16 $$\begin{array}{*{20}c} {SOL\left( {x,t} \right) = a_{s} + b_{s} \frac{n}{N}R_{s} } \\ \end{array}$$

where, $R_{s}$ is the solar shortwave radiation(MJ M^-2^ d^-1^), n is the actual sunshine time(hours), N is the time of day(hours), and $\frac{n}{N}$ is the relative sunshine time; The constants $a_{s} = 0.25$ and $b_{s} = 0.5$.

And the $\varepsilon \left( {x,t} \right)$ is calculated as follows:

17 $$\begin{array}{*{20}c} {\varepsilon \left( {x,t} \right) = T_{\varepsilon 1} \left( {x,t} \right) \times T_{\varepsilon 2} \left( {x,t} \right) \times W_{\varepsilon } \left( {x,t} \right) \times \varepsilon_{max} } \\ \end{array}$$

where, $T_{\varepsilon 1}$ and $T_{\varepsilon 2}$ are the stress factors of cold and heat, respectively; $W_{\varepsilon }$ is the water stress factor, reflecting the influence of water conditions; $\varepsilon_{max}$ is the maximum light use efficiency(gC MJ^-1^) under the optimal conditions, in this study, $\varepsilon_{max}$ is 0.389.

### Trend analysis

The Mann–Kendall nonparametric test and Sen’s slope estimator were used to analyze the trend of ESs in the QTP region. The Mann–Kendall method is widely used to analyze climatic and hydrological time series variation trends^77^. The advantage of the Mann–Kendall test is that it does not require the sample to follow a certain distribution, allows the existence of missing values, is not affected by a small number of outliers, and has strong quantitative ability^78^. The Mann–Kendall test is as follows:

18 $$\begin{array}{*{20}c} {S = \mathop \sum \limits_{i}^{n - 1} \mathop \sum \limits_{j = i + 1}^{n} sgn\left( {x_{j} - x_{i} } \right)} \\ \end{array}$$

For time series data, i.e., {x_1_, x_2_, …, x_n_}, n is the length of the data, and $sgn\left( {x_{j} - x_{i} } \right)$ is derived as:

19 $$\begin{array}{*{20}c} {sgn\left( {x_{j} - x_{i} } \right) = \left\{ {\begin{array}{*{20}c} { + 1,x_{j} - x_{i} > 0} \\ {0,x_{j} - x_{i} = 0} \\ { - 1,x_{j} - x_{i} < 0} \\ \end{array} } \right.} \\ \end{array}$$

In this study, we set the significance level of $\alpha = 0.05$, when $\left| Z \right| \le Z_{1 - \alpha /2}$ accepts the null hypothesis. Otherwise, the null hypothesis is rejected, and the trend is statistically significant.

20 $$\begin{array}{*{20}c} {Z = \left\{ {\begin{array}{*{20}l} \frac{S - 1}{{\sqrt {VAR\left( S \right)} }},&\quad S > 0 \\ 0,&\quad S = 0 \\ \frac{S + 1}{{\sqrt {VAR\left( S \right)} }},&\quad S < 0 \\ \end{array} } \right.} \\ \end{array}$$

21 $$\begin{array}{*{20}c} {VAR\left( S \right) = \left\{ {n\left( {n - 1} \right)\left( {2n + 5} \right) - \mathop \sum \limits_{j = 1}^{p} t_{j} \left( {t_{j} - 1} \right)\left( {2t_{j} + 5} \right)} \right\} \div 18} \\ \end{array}$$

where p is the number of nodes in the dataset and $t_{j}$ is the length of the nodes.

Sen’s slope estimator is an estimation method based on the median and its insensitivity to outliers^78^.

22 $$\begin{array}{*{20}c} {\beta = Median\left( {\frac{{x_{j} - x_{i} }}{j - i}} \right)} \\ \end{array}$$

### Trade-offs and synergy analysis

Synergies and trade-offs were used to describe the relationships among the ESs. A trade-off analysis was conducted to reflect the difference in ESs and their responses to climate change. Trade-offs are when ESs change in the opposite direction. Synergies are when ESs change in the same direction^79^. Correlation analysis is often used to evaluate trade-offs and synergies between ESs^2^. To analyze the trade-offs and synergies of ESs at different administrative and natural scales, we allocated the ES values at the 10 km (pixel), county and watershed scales by the “zonal statistic” module of ArcGIS 10.2, and conducted minimum–maximum normalization in R4.0.3 (www.R-project.com). To analyze the relationship between any two of the four ES types, the R package PerformanceAnalytics was adopted to measure the Spearman correlation matrix at different scales.

## Supplementary Information

Supplementary Information.
