# Supplementary material for: Dynamic characteristics and synergistic effects of ecosystem services under climate change scenarios on the Qinghai–Tibet Plateau
Source: Sci Rep. 2022 Feb 15;12:2540. doi: 10.1038/s41598-022-06350-0 (PMC8847625; doi:10.1038/s41598-022-06350-0)
Supplement: Supplementary file 1 — Supplementary Information. [file 41598_2022_6350_MOESM1_ESM.docx]

Supplementary data

Table S1. Data requirements and parameters for the InVEST model. WY-Water Yield, SR-Soil Retention, CS-Carbon Storage.

| **Data** | **source** | **Description** | **Resolution** | **InVEST model** |
| --- | --- | --- | --- | --- |
| LULC | Resource and Environment Science and Data Center, Chinese Academy of Sciences (RESDC) (<https://www.resdc.cn>) | Raster | 1km | WY,SDR,CS |
| Climate Data | climate change dataset(Su et al., 2017) | Raster | 1km | WY |
| Reference Evapotranspiration | Calculated by modified Hargreaves method |  | 1km | WY |
| DEM | Resource and Environment Science and Data Center, Chinese Academy of Sciences (RESDC) (<https://www.resdc.cn>) | Raster | 1km | SDR |
| Plant Available Water Fraction | International Soil Reference and Information Centre(ISRIC Data Hub) (<https://data.isric.org>) | Raster | 1km | WY |
| Watersheds/Sub-Watersheds | Resource and Environment Science and Data Center, Chinese Academy of Sciences (RESDC) (<https://www.resdc.cn>) | shape | - | WY,SDR |
| Depth to Root Restricting Layer | International Soil Reference and Information Centre(ISRIC Data Hub) (<https://data.isric.org>) | Raster | 1km | WY |
| Rainfall Erosivity Index | Calculated by monthly precipitation | Raster | 1km | SDR |
| Soil Erodibility | Calculated by content of sand,silt,clay and soil organic moisture | Raster | 1km | SDR |
| Carbon Pools | Literature review | - | - | CS |
| Biophysical Table | Literature review | CSV | - | WY,SDR |
| Z parameter | - | 9 | - | WY |
| Threshold Flow Accumulation | - | 1000 | - | SDR |
| Borselli k Parameter | - | 2 | - | SDR |
| Borselli IC0 Parameter | - | 0.5 | - | SDR |
| Max SDR Value | - | 0.8 | - | SDR |

Table S2. InVEST model biophysical parameter table

| **lucode** | **LULC_veg** | **ROOT_DEPTH** | **Kc** | **usle_c** | **usle_p** |
| --- | --- | --- | --- | --- | --- |
| 1 | 1 | 300 | 0.65 | 0.015 | 0.096 |
| 2 | 1 | 3000 | 1 | 0.006 | 0.01 |
| 3 | 1 | 300 | 0.65 | 0.03 | 0.01 |
| 4 | 0 | 1 | 1 | 1 | 1 |
| 5 | 0 | 1 | 0.2 | 1 | 1 |
| 6 | 0 | 1 | 0.2 | 1 | 1 |

Table S3. Area of land use types in QTP region(km2)

| **year** | | **cropland** | **forestland** | **grassland** | **water** | **built-up land** | **barren** |
| --- | --- | --- | --- | --- | --- | --- | --- |
| 1980 | | 18604 | 272270 | 1504337 | 118346 | 1137 | 652005 |
| 1990 | | 18753 | 272272 | 1508141 | 102123 | 1221 | 664189 |
| 1995 | | 18791 | 272297 | 1507821 | 101845 | 1217 | 664748 |
| 2000 | | 19145 | 271855 | 1507304 | 102018 | 1324 | 665073 |
| 2005 | | 19040 | 271651 | 1506680 | 102733 | 1590 | 665025 |
| 2010 | | 19057 | 271728 | 1506516 | 103032 | 1738 | 664648 |
| 2015 | | 18945 | 271620 | 1505040 | 104718 | 2302 | 664097 |
| 2030 | RCP2.6 | 18821 | 272036 | 1505698 | 104914 | 2478 | 662770 |
|  | RCP4.5 | 19124 | 272036 | 1505698 | 104611 | 2478 | 662770 |
|  | RCP8.5 | 19124 | 272036 | 1505698 | 104611 | 2478 | 662770 |
| 2050 | RCP2.6 | 19190 | 272344 | 1505207 | 105410 | 2918 | 661648 |
|  | RCP4.5 | 19190 | 272344 | 1505207 | 105410 | 2918 | 661648 |
|  | RCP8.5 | 19190 | 272344 | 1505207 | 105410 | 2918 | 661648 |
| 2100 | RCP2.6 | 19351 | 273112 | 1503570 | 108347 | 4400 | 657937 |
|  | RCP4.5 | 19351 | 273112 | 1503570 | 108347 | 4366 | 657971 |
|  | RCP8.5 | 19351 | 273112 | 1503570 | 108347 | 4331 | 658006 |


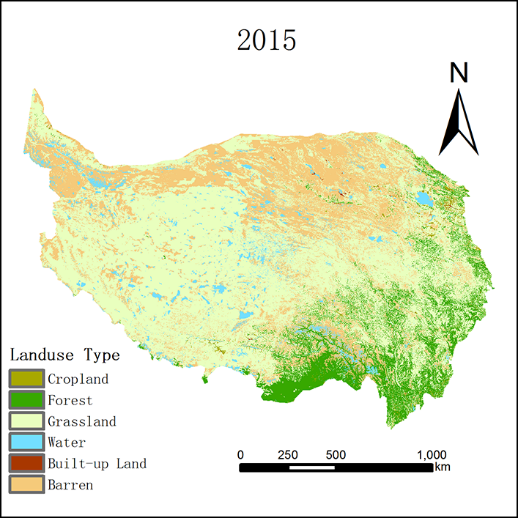


Figure S1. land use map in 2015. The map was created using ArcMap 10.2, URL: http://www.esri.com.


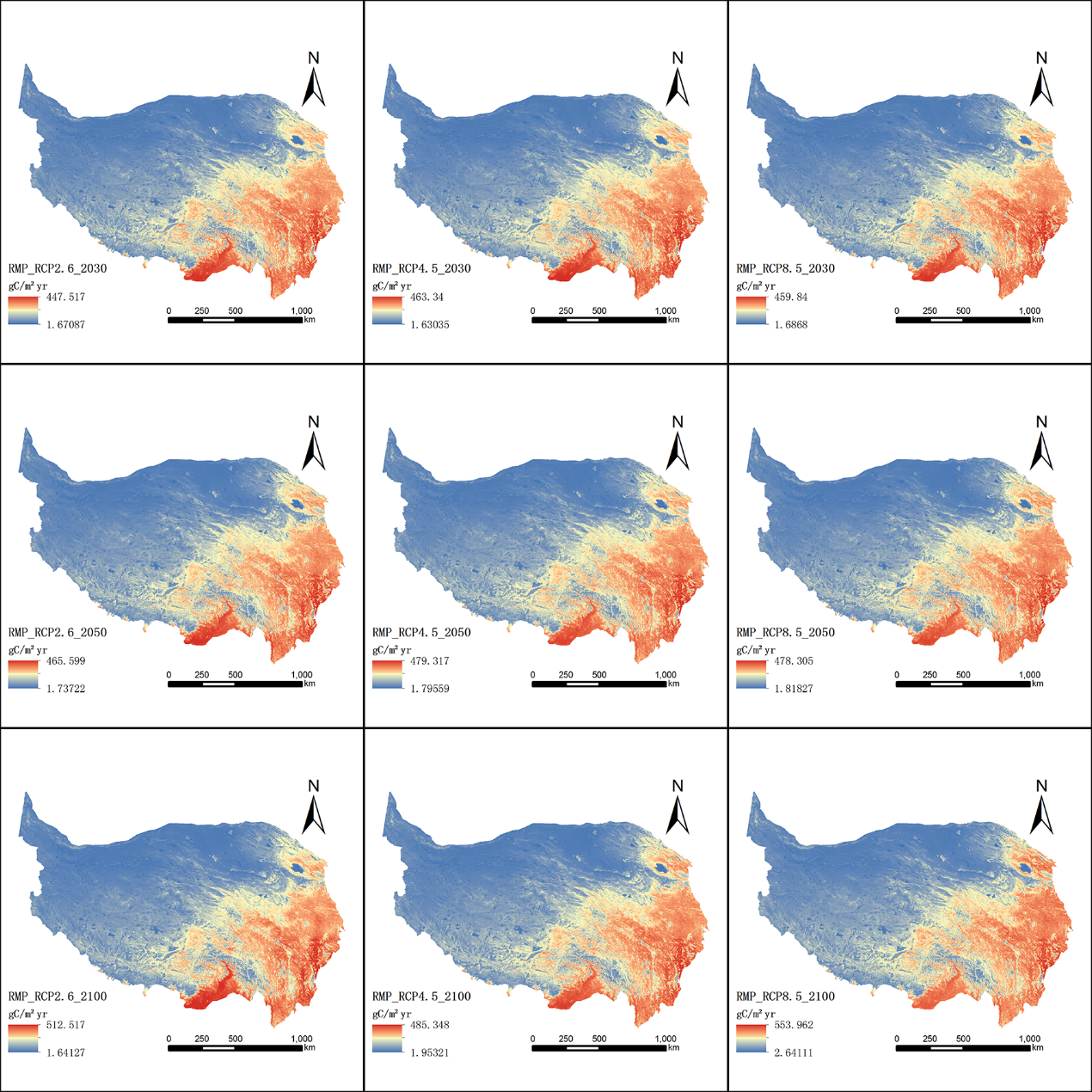


Figure S2. Spatial pattern of raw material provision under RCPs scenarios in 2030,2050 and 2100. The map was created using ArcMap 10.2, URL: http://www.esri.com.


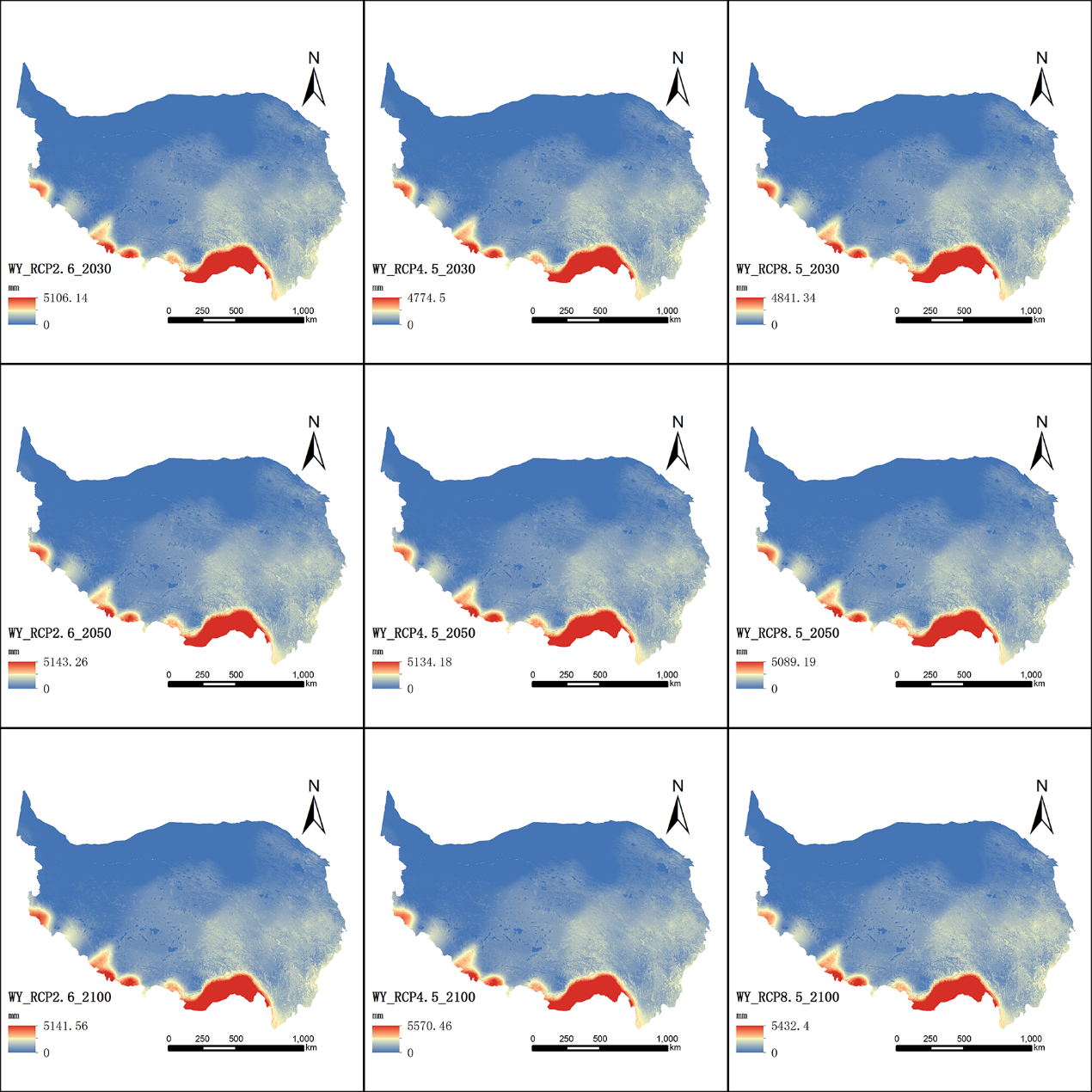


Figure S3. Spatial pattern of water yield under RCPs scenarios in 2030,2050 and 2100. The map was created using ArcMap 10.2, URL: http://www.esri.com.


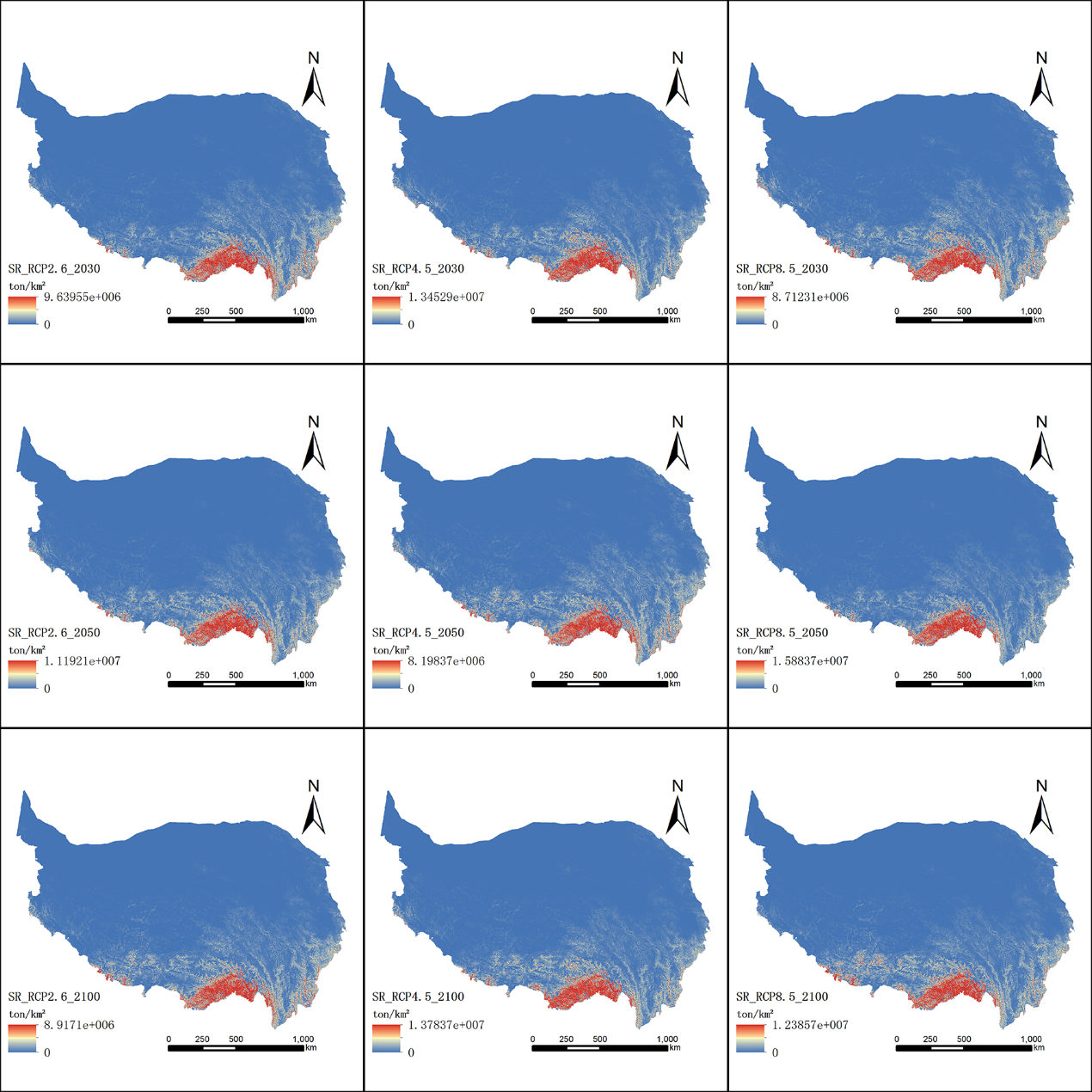


Figure S4. Spatial pattern of soil retention under RCPs scenarios in 2030,2050 and 2100. The map was created using ArcMap 10.2, URL: http://www.esri.com.


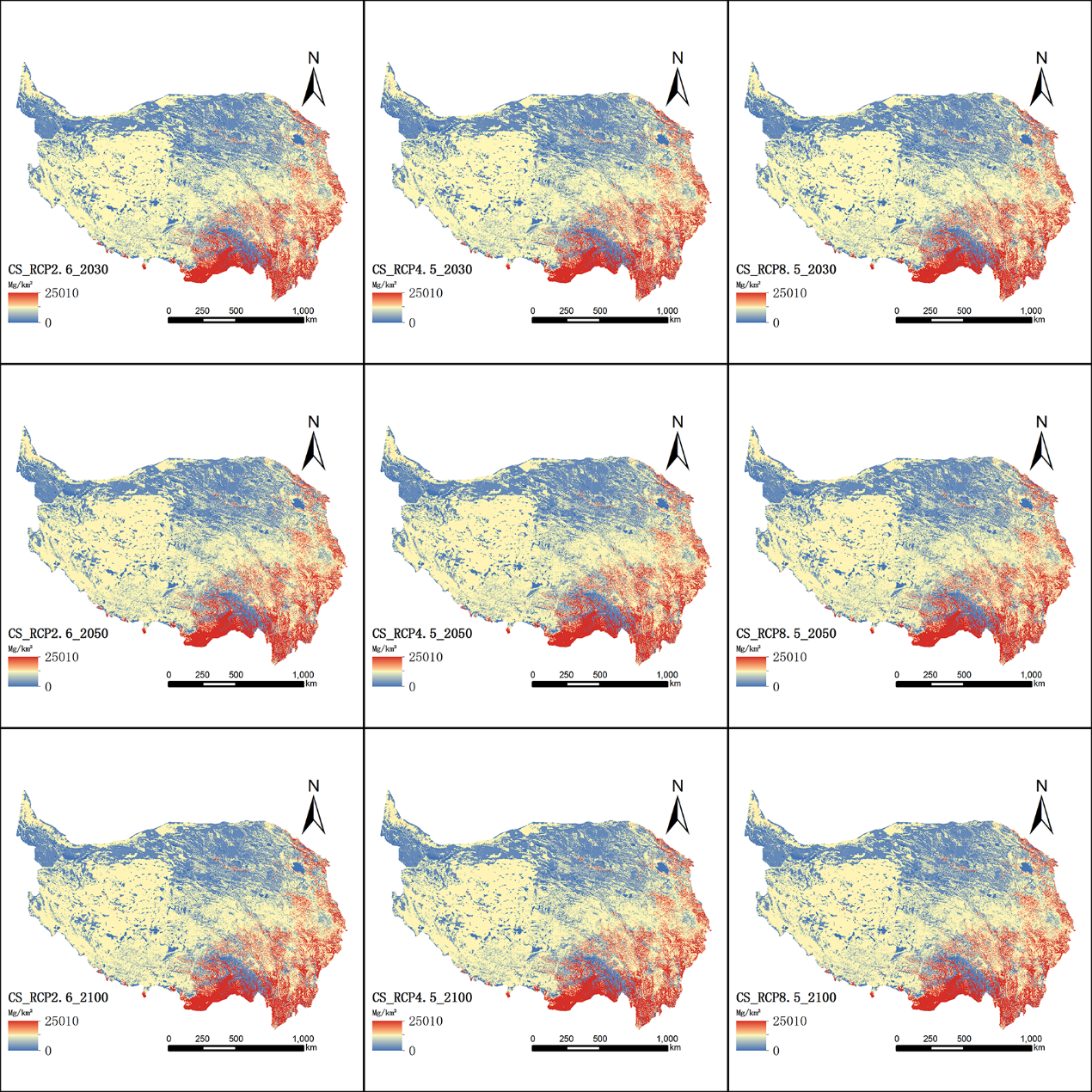


Figure S5. Spatial pattern of carbon storage under RCPs scenarios in 2030,2050 and 2100. The map was created using ArcMap 10.2, URL: http://www.esri.com.


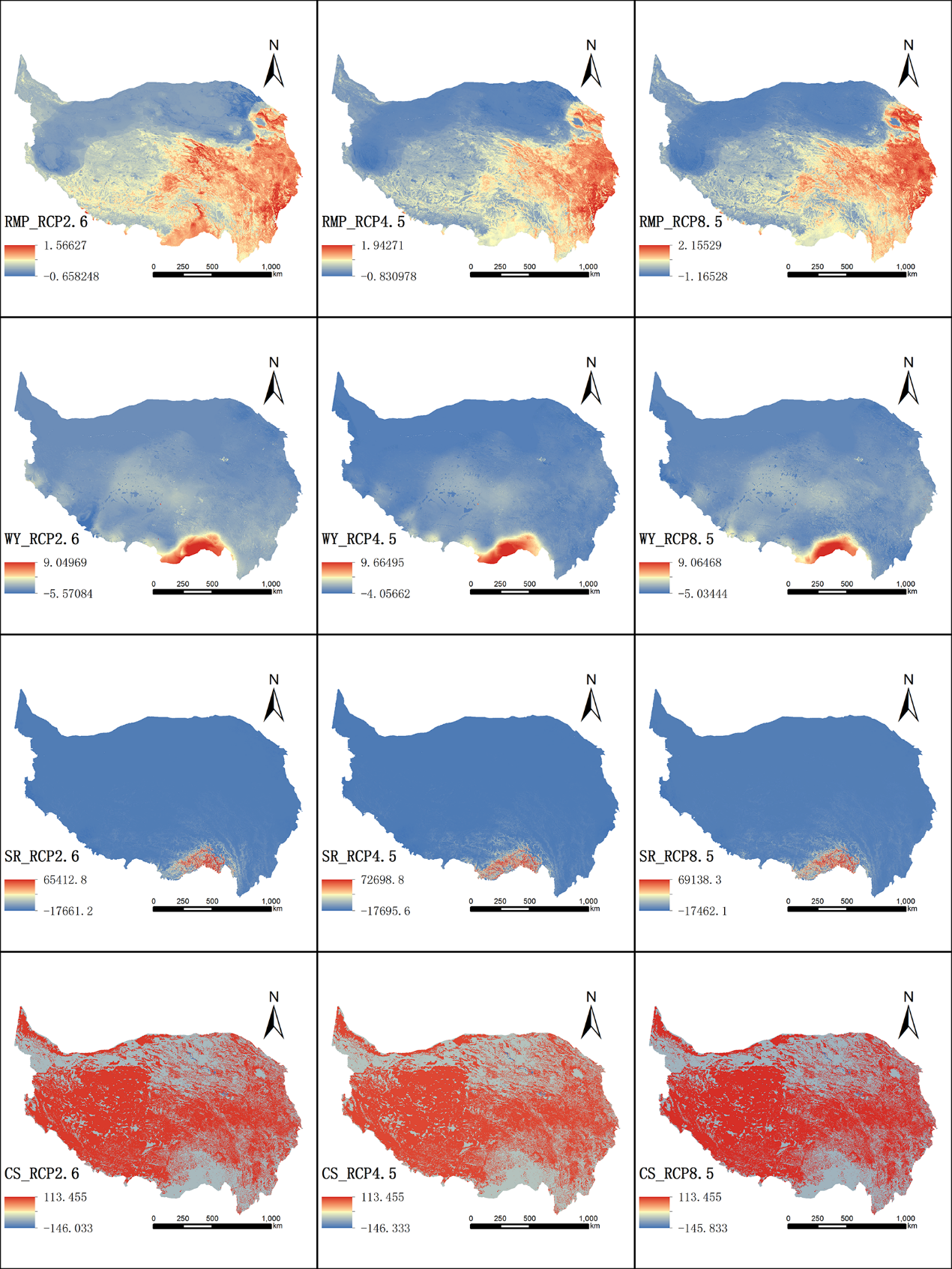


Figure S6. The Sen’s slop analysis of ESs. The map was created using ArcMap 10.2, URL: http://www.esri.com.


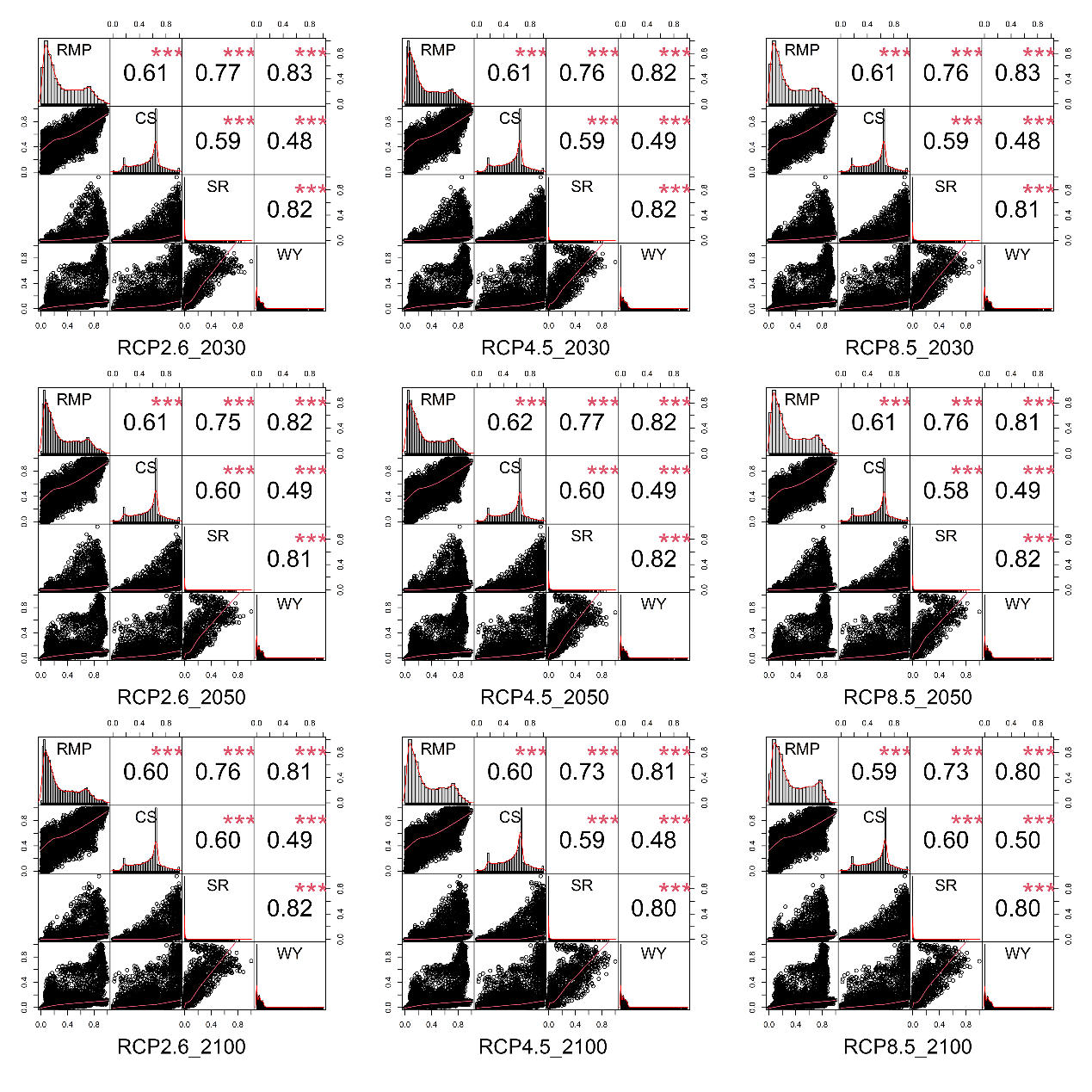


Figure S7. Trade-offs and synergies between ecosystem services at 10km scale in 2030,2050 and 2100 under RCPs scenarios. The number above the diagonal represents Spearman’s correlation coefficient. RMP: raw material provision; CS: carbon storage; SR: soil retention; WY: water yield. (*** for p<0.001)


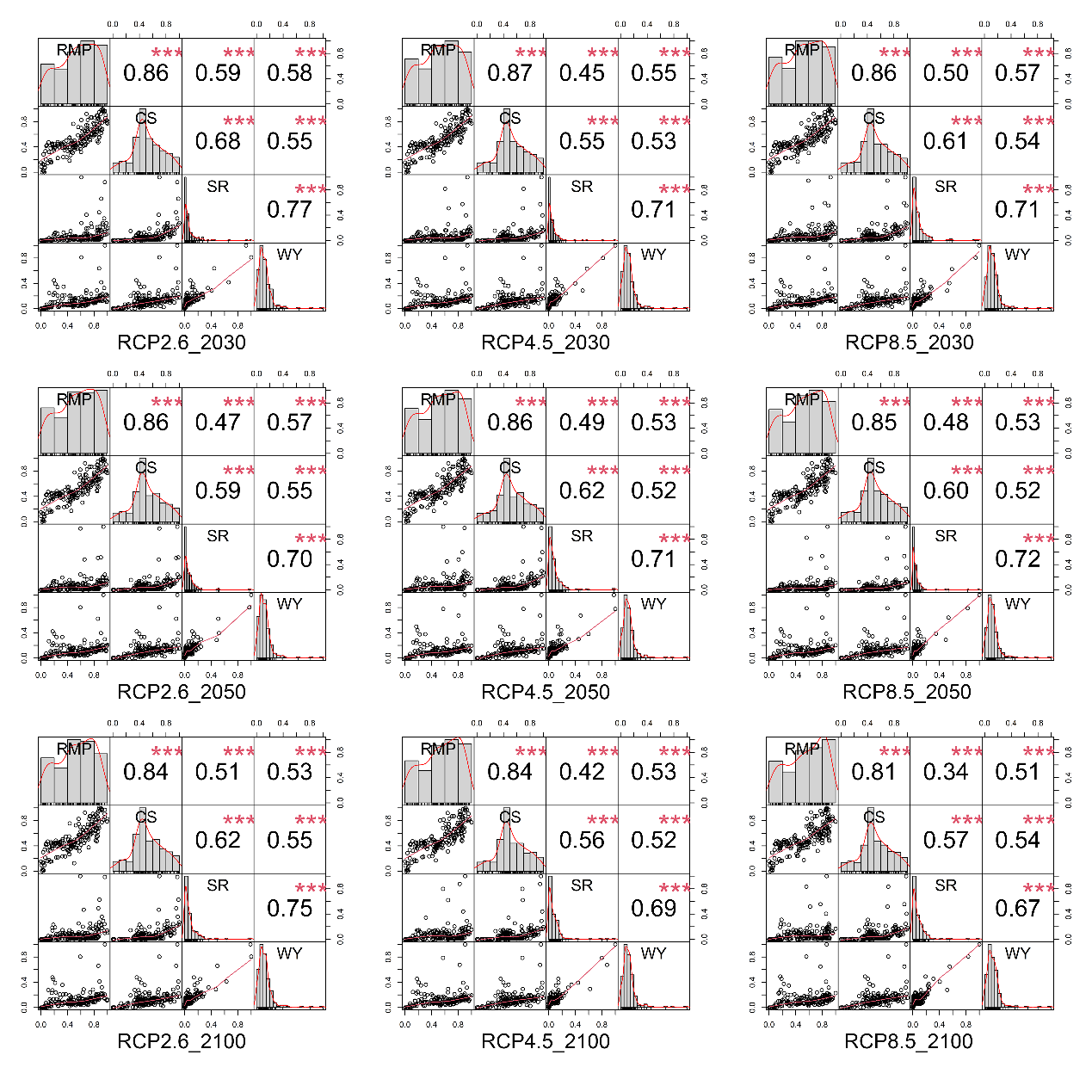


Figure S8. Trade-offs and synergies between ecosystem services at county scale in 2030,2050 and 2100 under RCPs scenarios. The number above the diagonal represents Spearman’s correlation coefficient. RMP: raw material provision; CS: carbon storage; SR: soil retention; WY: water yield. (*** for p<0.001)


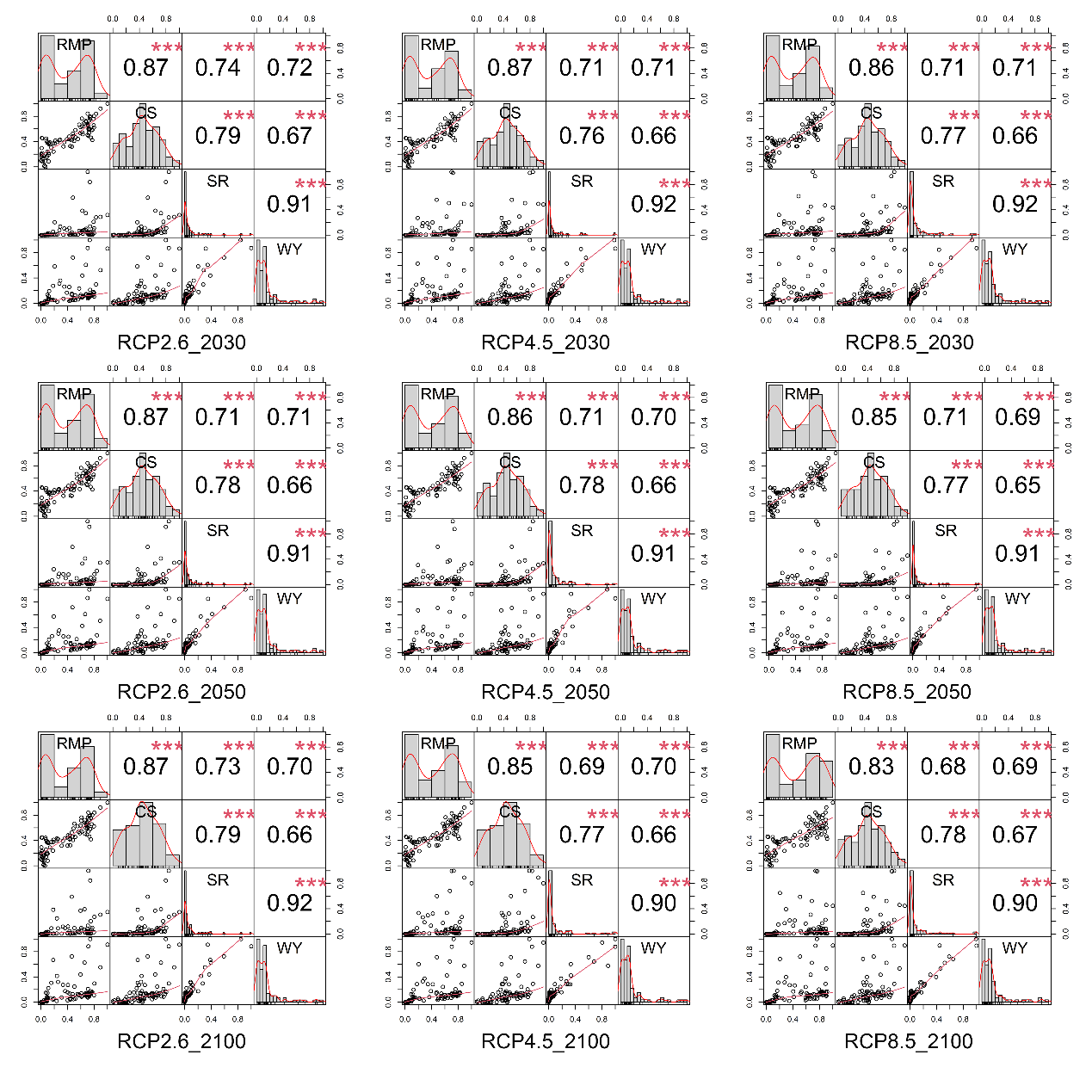


Figure S9. Trade-offs and synergies between ecosystem services at watershed scale in 2030,2050 and 2100 under RCPs scenarios. The number above the diagonal represents Spearman’s correlation coefficient. RMP: raw material provision; CS: carbon storage; SR: soil retention; WY: water yield. (*** for p<0.001)

**Reference**

Su BD, Huang JL, Zeng XF, Gao C, Jiang T. Impacts of climate change on streamflow in the upper Yangtze River basin. Climatic Change 2017; 141: 533-546.
